# Effect of the haematocrit layer geometry on *Plasmodium falciparum *static thin-layer *in vitro *cultures

**DOI:** 10.1186/1475-2875-7-203

**Published:** 2008-10-08

**Authors:** Jordi Ferrer, Marina D Rosal, Jaume M Vidal, Clara Prats, Joaquim Valls, Esperanza A Herreros, Daniel López, Domingo Gargallo

**Affiliations:** 1 Escola Superior d'Agricultura de Barcelona, Campus del Baix Llobregat, Departament de Física i Enginyeria Nuclear, Universitat Politècnica de Catalunya, Avda. del Canal Olímpic 15, 08860 Castelldefels, Spain; 2 Drug Discovery Biology Group, Diseases of the Developing World Center, of GlaxoSmithKline R&D, Tres Cantos, Madrid, Spain

## Abstract

**Background:**

*In vitro *cultivation of *Plasmodium falciparum *is usually carried out through the continuous preservation of infected erythrocytes deposited in static thin layers of settled haematocrit. This technique, called the candle-jar method, was first achieved by Trager and Jensen in 1976 and has undergone slight modifications since then. However, no systematic studies concerning the geometry of the haematocrit layer have been carried out. In this work, a thorough investigation of the effects of the geometric culturing conditions on the parasite's development is presented.

**Methods:**

Several experimental trials exploring different settings have been carried out, covering haematocrit layer depths that ranged from 6 mm to 3 mm and separation between the walls of the culturing device that ranged from 7.5 mm to 9 mm. The obtained results have been analysed and compared to different system-level models and to an Individual-Based Model.

**Conclusion:**

In line with the results, a mechanism governing the propagation of the infection which limits it to the vicinity of the interface between the haematocrit layer and the culture medium is deduced, and the most appropriate configurations are proposed for further experimental assays.

## Background

Malaria parasites were first preserved *in vitro *without loss of infective viability by Pavanand *et al *[1]. Continuous culture of viable *Plasmodium falciparum *schizogonic stages was first achieved soon after by means of cultures that maintained infected red blood cells (RBCs) settled in a thin deposit under controlled conditions (static cultures). Trager and Jensen developed two types of static cultures: a system with continuous medium replacement, and a system with discrete daily medium renewal, called the candle-jar method [2]. The protocols then reported have been widely studied, and many modifications have been tried [3-6]. During the 1980s and the 1990s, the possibility of establishing suspended cultures was also explored [7,8], and several semi-automated methods for continuous parasite culture were also proposed [9,10]. Static cultivation of infected RBCs prevails as the most widespread parasite reservoir for clinical and pharmacological purposes.

The candle-jar method has been standardized for general use and is currently employed with slight modifications [11], yet several aspects regarding how the culturing methodology affects the development and growth of the parasite are not yet fully understood. This work is focused on two of these features:

*i) In vitro *parasite growth and survival in static cultures is dramatically hindered at high parasitaemias (above 10%). According to the scientists at KEMRI [11], this upper threshold can be raised to higher parasitaemias by increasing the share of culture medium (cMCM) per RBC. However, such limitation does not exist for suspended cultures with agitated medium [7], which suggests that the lack of suitable conditions for parasite development is not due to the global exhaustion of the culture medium, but rather to the local inhospitableness of the surroundings of the parasite caused by diffusive limitations of the haematocrit layer or by other restrictions on any relevant local transport phenomenon, such as parasite spread.

*ii) *Static cultures are often referred to as 'thin-layer cultures', yet the exact meaning of 'thin' is not specified. In the MR4 protocols alone, haematocrit layer depths ranging from 0.8 mm to 0.15 mm are proposed without distinction, and the range of proposed depths widens when more sources are consulted. Such differences in haematocrit thickness are at least one order of magnitude greater than the RBC's characteristic length (8·10^-3 ^mm), and may be highly significant if local transport phenomena are relevant to the spreading of the infection. The authors could not find systematic studies supporting the choice of the proposed thickness in the literature, but the published recommendations reflect the experience accumulated since cultivation was first achieved.

The scope presented suggests that macroscopic physical and geometric constraints on the culturing system play an important role in the *in vitro *parasite development. This role must be initially assessed as the groundwork for acquiring predictive insight of the system and improving current culturing methods.

In this study, the effect of the haematocrit layer dimensions on static *P. falciparum in vitro *cultures is analysed. To do this, three series of experiments were carried out by the Experimental Microbiology Group (EMG) at the Drug Discovery Centre for Diseases of the Developing World GlaxoSmithKline R&D (Tres Cantos, Madrid). The trials compare different base surfaces, culture volumes and shapes for the culture vials. Experimental data were used to check different system-level population models and to propose some mechanisms governing the system at a cellular level compatible with the observed behaviour of the whole culture. The consistency of the proposed individual-level models was tested through a bottom-up approach. In order to explore this avenue, INDISIM (INDividual DIScrete SIMulation), an Individual-Based Model (IBM) specifically designed to deal with microbial communities, was used [12].

## Methods

### General experimental procedure

*Plasmodium falciparum *3D7A infected RBCs static *in vitro *cultures were raised under similar culturing conditions, and could be distinguished by the geometric characteristics of the haematocrit layer, once the RBCs had settled. The cultures were prepared according to the MR4 protocols [11].

*Plasmodium falciparum *cultures were carried out in complete culture medium (cMCM) (RPMI 1640 medium supplemented with 25 mM HEPES, 10% human serum and 0.15 mM Hypoxanthine). Red blood cells supplied by the Spanish Red Cross were added at 5% of haematocrit value (H = 5 0 ± 0.4%). The age of red blood cells used in this study was less than 30 days. Red blood cells were washed three times with RPMI prior to use and washed cells were stored at the most for seven days at 4°C. Initial parasitaemia was set to *I*_0 _= 0.5%. Cultures were incubated at 37°C in low oxygen atmosphere (5% O_2_, 5% CO_2_, 90% N_2_). Dilution of cultures and medium renewal were performed every 48 h in order to reach initial parasitaemia. Parasitaemia was measured through optical microscopy of Giemsa-stained thin blood smears sampled from the culture every 24 hours, counting parasites appearing in 2500 red blood cells. Output data were statistically treated using parametric and non-parametric statistics.

Two different kinds of experiments were performed through three different series (*B*, *W *and *P*), namely:

*i) *trials covering a range of base areas after fixing both the haematocrit layer volume and depth: *P *series were carried out to evaluate the effect of the walls of the culture device. They were set in 90 mm diameter glass Petri dishes that had been split into detached subregions by gluing glass separators onto the plate base surface. Different distances between the glass separators (*L*) were checked. Each subregion was evaluated separately and a control monitoring of a subregion of the culturing device with fixed base surface was performed for each trial.

*ii) *trials varying the haematocrit layer depth (*HLD*) by modifying both the surface base and the total culture volume: *B *and *W *series were carried out to assess the effect of the *HLD *and of the total culture volume. The former were carried out in 50 mm diameter flat-bottomed glass bottles using large culturing volumes (from 2 ml to 100 ml), while smaller culture volumes (from 1 ml to 10 ml) set in 3.5 mm diameter plastic wells were used in the *W *series. *HLD *was calculated according to the model presented in Section 2.2.3.

Output measurements of the total parasitaemia were performed daily for both kinds of experiments. The infection growth ratio at 48 hours was calculated from the measured data. Geometric characteristics of the experimental sets are presented in Tables 1 and 2.

**Table 1:** Experimental *L *culture trials

***Trial name***	*V *(*ml*)	*V*_*RCB *_(*μl*)	*L *(*cm*)	*S (cm*^2^)	*V*_*H *_(*μl*)	*HLD *(*mm*)	*p *(%)	*R*_48_
**P1**	16.5	825	0.75	6.8	129.2	0.19	0.4 ± 0.2	0.6 ± 0.8
**P2**	16.5	825	1	9.0	171.0	0.19	0.6 ± 0.2	1.3 ± 0.8
**P3**	16.5	825	2	16.0	304.0	0.19	0.7 ± 0.4	2.2 ± 2.1
**P4**	16.5	825	4	32.0	608.0	0.19	0.9 ± 0.3	3.3 ± 1.6
**P5**	16.5	825	9	63.6	1208.4	0.19	1.2 ± 0.5	4.1 ± 1.5

Macroscopic geometric characteristic parameters and experimental results of the trials carried out to assess the effect of the distance to the walls of the device on culture parasite development. P5 is the control plate with no internal walls. *V *refers to the measured total culture volume, *V*_*RBC *_refers to the calculated volume of packed RBCs and *V*_*H *_refers to the calculated volume of the haematocrit layer. *L *refers to the measured distance between separators and *S *denotes the calculated base surface. *HLD *is the calculated depth of the haematocrit layer. *p *is the average observed parasitaemia and *R*_48 _is the calculated growth ratio at 48 hours.

**Table 2:** Experimental *HLD *culture trials

***Trial name***	*V *(*ml*)	*V*_*RBC*_(*μl*)	*D *(*cm*)	*S *(*cm*^2^)	*V*_*H*_(*μl*)	*HLD *(*mm*)	*P *(%)	*R*_48_
**W1**	1.0	49.4	3.5	9.62	73.73	0.060. ± 0.015	1.5 ± 1.2	5.2 ± 1.8
**W2**	1.5	74.1	3.5	9.62	110.60	0.09 ± 0.02	1.6 ± 1.1	5.4 ± 1.9
**W3**	3.1	155.5	3.5	9.62	194.03	0.19 ± 0.05	1.7 ± 1.3	6 ± 2
**W4**	5.8	290	3.5	9.62	232.09	0.34 ± 0.08	1.5 ± 1.0	4.9 ± 1.0
**W5**	10.0	500	3.5	9.62	388.06	0.59 ± 0.15	1.4 ± 1.0	5 ± 2
**B1**	2.6	130	3.1	7.54	432.84	0.18 ± 0.05	1.8 ± 1.2	6.3 ± 1.2
**B2**	5.2	260	3.3	8.55	432.84	0.34 ± 0.08	1.7 ± 1.2	6.4 ± 0.9
**B3**	5.8	290	3.4	9.08	746.27	0.32 ± 0.08	1.7 ± 1.6	7 ± 3
**B4**	10.1	506.5	3.6	10.1	755.97	0.56 ± 0.14	1.4 ± 0.8	5.1 ± 0.8
**B5**	10.4	520	3.5	9.62	776.12	0.60 ± 0.15	1.4 ± 1.0	5.1 ± 1.8
**B6**	20.8	1040	3.9	11.9	1552.24	1.0 ± 0.2	1.1 ± 1.5	3.3 ± 0.7
**B7**	26.0	1300	4.1	13.2	1940.30	1.1 ± 0.3	1.0 ± 0.4	3.0 ± 0.5
**B8**	51.5	2074	5	19.6	3841.79	1.5 ± 0.4	0.7 ± 0.2	1.6 ± 0.3
**B9**	68.6	3432	5	19.6	5122.39	2.0 ± 0.5	0.50 ± 0.19	1.6 ± 0.4
**B10**	74.1	3705	5	19.6	5529.85	2.2 ± 0.5	0.4 ± 0.2	1.0 ± 0.2
**B11**	98.8	4940	5	19.6	7373.13	2.9 ± 0.7	0.3 ± 0.16	0.8 ± 0.4

Macroscopic geometric characteristic parameters and experimental results of the trials performed to assess the effect of *HLD *on the culture development. *V *refers to the total culture volume, *V*_*RBC *_refers to the calculated volume of packed RBCs, and *V*_*H *_refers to the calculated volume of the haematocrit layer. *D *refers to the diameter of the haematocrit layer deposit and *S *denotes the calculated base surface. *HLD *is the calculated depth of the haematocrit layer, *p *is the average of the observed parasitaemias and *R*_48 _is the calculated growth ratio at 48 h. *W *refers to cultures in 6-well plastic plates and *B *to cultures in 5 cm diameter flat-bottom glass bottles. *W *assays with volumes lower than 1.0 ml have not been presented because they did not develop well, due to the effects of surface tension.

### Geometric macroscopic model of the culture system and haematocrit layer

Accurate measurements of some of the relevant geometric characteristics of the culture system can not be successfully taken with the desired precision, and they must be estimated, calculated or measured indirectly. Several ad-hoc assumptions are implicitly accepted when calculating these values.

#### RBC concentration in the haematocrit deposit

Cultures were prepared by diluting packed RBCs into cMCM to 5% haematocrit concentration in volume. RBCs form a deposit at the bottom of the culturing device, creating a stable haematocrit layer. The fraction of the haematocrit culture layer occupied by RBCs (*f*) was indirectly measured in the custom cultures in T-flasks and in flat-bottomed test tubes by measuring the real and observed volume of the haematocrit layer: *f *= 0.89 ± 0.08.

It may be assumed that this fraction remains constant throughout the haematocrit layer; in other words there is no compacting due to hydrostatic effects. This 'lack of compacting' hypothesis yields an average concentration of RBCs throughout the entire haematocrit layer, rather than a stratified deposit, and can be assumed because the net weight of the settled RBCs over any single cell is negligible when compared both to the cellular stiffness [13,14] and to the RBC-RBC interactions [15]. Under this assumption, it is possible to define a simple hard-sphere model for the haematocrit layer: RBCs are placed in an ordered grid composed of 5 μm-sided cubic cells, arranged in one RBC per cubic cell, at most.

This model is a great simplification, yet it allows the estimation of the *HLD *given the culture volume. The real structure of the haematocrit layer is expected to be more disordered and considerably more difficult to model, as RBCs aggregate to form mesoscale structures, such as 'rouleaux' and 'rosettes'. Such structures imply both the accumulation of RBCs in dense conglomerations and the creation of large interstitial cavities between aggregates.

#### Intercellular interactions and shape of the haematocrit layer

Both healthy and infected RBCs are subject to some extent to cell-to-cell attraction interactions: rouleaux and other kinds of RBC-RBC aggregates are formed due to the presence of proteins in plasma, which is greater in sepsis and under stressful conditions [16]. The average RBC-RBC adhesion energy per unit surface (*g*_*RBC*_) is estimated to be $\gamma_{RBC}=\text{1}\cdot {\text{10}}^{-\text{4}}\frac{N}{m}$. Rosettes are cellular clusters that usually bind a few RBCs to a single infected RBC (IRBC), but they may contain up to 20 IRBCs and 50 uninfected RBCs during cerebral malaria and in *in vitro *cultures with high parasitaemias [17]. The authors could not find any estimation of the rosette binding energy in the literature, but it is obviously greater than RBC-RBC adhesion energies. Otherwise rosettes would not be observed.

**Figure 1:**
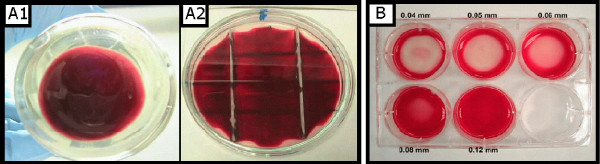
**Different observed shapes of the haematocrit layer. **A) Glass culturing devices produce a convex meniscus. A1: flat bottomed bottles used in the *B *series. A2: Petri dishes with glass separators used in the *P *series. B) Plastic culturing devices produce a concave meniscus, as can be observed in the 6-well plates used in the *W *series.

The intercellular attraction produces a net inward pull on the cells on the border of the haematocrit. The interface between the haematocrit layer and the free culture medium shows a property similar to the surface tension of liquids [18]. In consequence, under certain geometric conditions, the layer is not strictly a thin flat deposit that covers all the bottom of the culturing device, but rather resembles a sessile drop. The shape of the layer depends on the ratio between the intercellular attractive forces and the RBC adhesion to the walls of the culturing device. For small volumes of packed RBCs, the haematocrit layer covers only a fraction of the total available surface at the bottom of the culturing device, and different shapes can be observed for wettable (polymer surfaces) and non-wettable materials such as glass surfaces (Figure 1).

The analogy with liquids can be used to define the capillary length ($L_{c}=\sqrt{\frac{\gamma }{g\Delta \rho }}$), the haracteristic length scale in which intercellular interactions are comparable to gravitational energy.

*L*_*C *_defines the extent to which the effects of the surface tension must be taken into account. For *in vitro *cultures, $\Delta \rho =\text{140}\frac{Kg}{m^{\text{3}}}$ is the difference in density between an average RBC and the culturing medium, and is $g=\text{9}\text{.8}\frac{m}{s^{\text{2}}}$ gravity. By considering just the RBC-RBC interactions $\gamma_{RBC}=\text{1}\cdot {\text{10}}^{-\text{4}}\frac{N}{m}$[15], a minimum threshold for the capillary length can be defined: *L*_*c *_= 0.27 *mm*. In this context, *L*_*C *_can be associated with the maximum depth of an unbounded sessile drop [19], meaning the maximum depth that the haematocrit layer can reach when it is settled in the bottom of a culturing device without being in contact with its lateral walls. It can also be associated with the area of the region affected by the curve of the meniscus, as, for instance, the fraction of the layer which is affected by the presence of the walls of the culturing device when the layer covers the whole bottom surface (*L*_*EXC*_). It must be pointed out that this calculated value for *L*_*C *_is expected to be an underestimation of the value calculated from experimental observations, because the present estimation does not account for the strengthening of intercellular interactions (adhesiveness) caused by the infection.

### Estimation of the experimental haematocrit layer depth

The capillary length determines the shape and depth of the haematocrit deposit for very small cultures. The usual culturing conditions imply spatial scales much larger than the capillary length and thus surface tension effects may be neglected and the haematocrit layer may be considered a flat thin bed.

Under this assumption, haematocrit layer depth (*HLD*) is calculated from the total culture volume (*V*), haematocrit concentration (*H*) and fraction of the haematocrit layer occupied by RBCs (*f*) (Equation 1).

(1) $$HLD=\frac{V\cdot H}{S\cdot f}$$

The calculated *HLD *is used from now on to characterize the effect of the haematocrit depth on parasite development in static *in vitro *cultures

## Results

Experiments reveal two geometric characteristics of the haematocrit layer that affect the *in vitro *development of the parasite: depth (*HLD*) and separation between walls of the culturing device (*L*). Culture characteristics and observed results for *W*, *B *and *P *trials are presented in Tables 1 and 2.

The observed measurements throughout each trial show a strong time correlation, as a consequence of both the parasite infection cycle and the external manipulation of the culture system. In fact, each single trial can be regarded as the evolution of a single culture, thus characterized by the average parasitaemia throughout the whole culture trial (*p*), or as a set of replicas of the evolution of the culture between two subsequent sub-cultivations, thus characterized by the average growth ratio (*R*_48_). The latter approach seems to be more appropriate in building geometric models to better understand the evolution of the culture, because the cycles throughout each trial show a similar behaviour and no significant long-term evolution can be distinguished in a scale broader than the subcultivation cycles. Thus the data sets from each trial comprise a variable amount of the observed growth ratios between two successive subcultivations. Further, a Jarque-Bera (JB) test shows that data sets from any single trial do not come from a normal distribution, so the observed statistical dispersion of the measurements of the parasitaemia must not be mistaken for a standard deviation from the average value. The calculated growth ratios barely fulfil the JB test requirement with 90% significance. Thus, the comparison of the data from each of the trials within a series must be tackled using non-parametric statistics.

Experimental results were also compared to geometric system-level models and to bottom-up approaches (IBMs). Comparison between theoretical outcomes and experimental results were carried out assuming that both data sets were sampled from normal distributions.

### Effect of the walls of the culturing device (*L *model)

**Figure 2:**
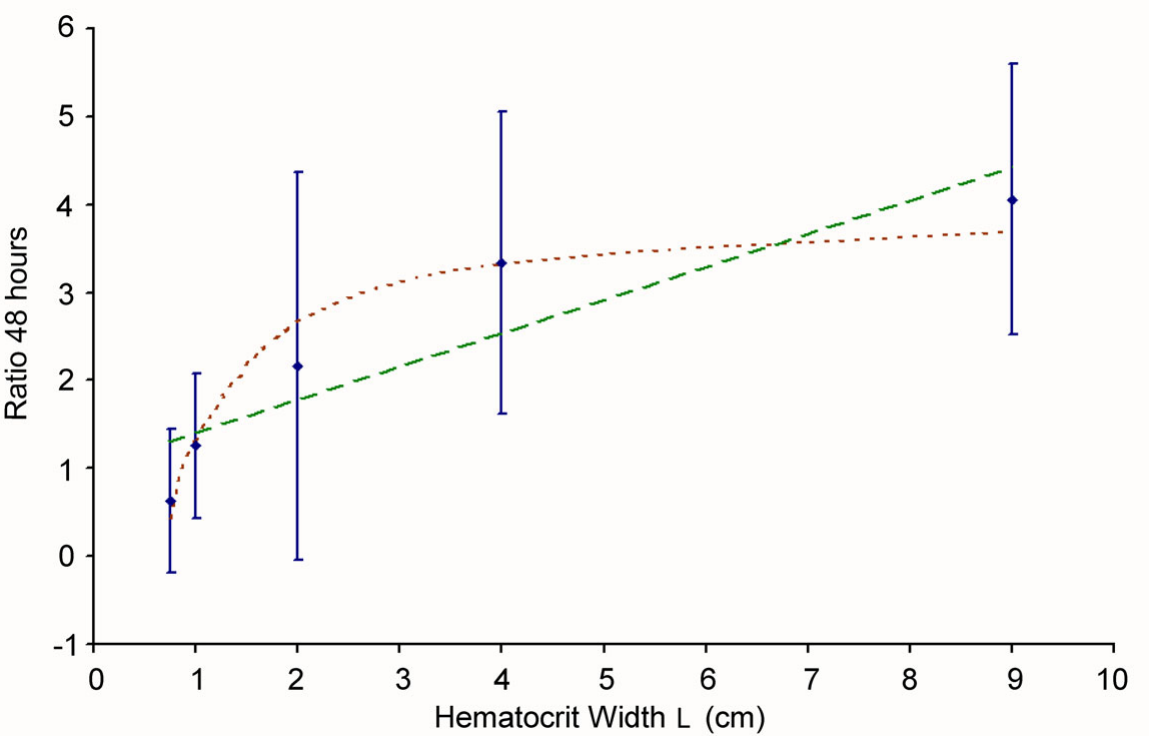
**Observed dependence of the average growth ratio at 48 h on the distance between separators (*L*).** Dots with error bars represent the observed data. Dashed line denotes the best linear fit. Dotted line denotes the best inversely linear fit (WS model).

The first aim was to check whether the base surface (*S*) of the haematocrit layer was a factor affecting the *in vitro *development of the parasite. Prior comparisons showed that the base surface was not significant in the range of the usual culture values [11], but the effect of the extension of the haematocrit turns out to be important when the separation between the walls (*L*) of the culturing device diminishes. The significance of the distance between walls can be assessed using a non-parametric statistic such as the Kruskal-Wallis (KW) test. Data from different trials show that the development of the parasite is clearly hindered by very small separations between walls, with 99% confidence (p-value = 0.00014). However, the functional dependence of this hindrance on *L *can not be significantly determined: the observed average growth ratio at 48 hours from the *P *series can be fitted to the same degree of confidence both to a linear regression and to an inversely linear regression (Figure 2).

Best fits are *R*_*linear*_(*L*) = 1.02+0.38·*L *and *R*_*inversely linear*_(*L*) = 4.01 + $\frac{\text{2}\text{.68}}{L}$, respectively.

The chi-square test applied to the experimental data series shows that either dependence is supported by statistical significance of approximately 80% (sic.: there is a 20% chance of obtaining equal or better fitting results assuming that experimental data do not come from the distribution proposed by the model).

**Figure 3:**
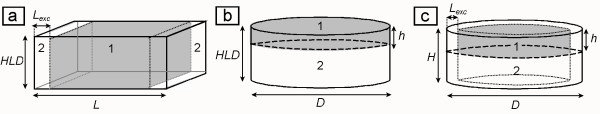
**Depiction of the whole system model of the haematocrit layer.** a) Schema of the culture system according to the model used to tackle *P *trials. *HLD *indicates haematocrit layer depth, *L *stands for the separation between walls of the culturing device and *L*_*EXC *_represents the extent of the exclusion region, where the spread of the infection is hindered. The shaded subregion (1) indicates the fraction of the haematocrit layer where the rate of infection spreading is high. b) Schema of the culture system according to the model used to tackle *B *and *W *trials. *HLD *indicates the haematocrit layer depth, *D *stands for the diameter of the haematocrit layer and *h *represents the region where the spread of the infection is not hindered. The shaded subregion (1) indicates the fraction of the haematocrit layer where the rate of infection spreading is high. c) Schaema of the model to tackle all the geometric effects simultaneously. The propagation of the infection is hindered both by the walls of the culturing device and by diffusive limitations. The shaded subregion (1) indicates the fraction of the haematocrit layer where the rate of infection spread is high.

The inversely linear dependence can be explained by means of a whole-system model that considers an exclusion region in the vicinity of the walls of the culturing device where the infection cannot progress (see Figure 3a). Under this assumption, the expected average growth ratio of a culture as a function of the separation between walls (*R*_48_(*L*)) is described by curve 2:

(2) $$R_{\text{48}}(L)=A_{1}\frac{{\text{2L}}_{EXC}}{L}+A_{2}\text{(1}-\frac{{\text{2L}}_{EXC}}{L})$$

where *A*_2 _is the growth ratio in the exclusion region (*L*_*EXC*_), and *A*_1 _is the bulk growth ratio (*A*_1 _> *A*_2_). The parameters that best fit this curve to the experimental data set are shown in Table 3. According to this fit, the exclusion region would be spread over approximately *L*_*EXC *_= 2.5 *mm*. Many microscopic mechanisms may be speculated as being responsible for creating this exclusion region (e. g., limitations on the spread of the metabolic waste products, hindered propagation of the parasite due to the existence of a meniscus) but it is not possible to discriminate among them with the current information. In particular, the observed exclusion region is consistent with the expected exclusion region induced by the capillary length (*L*_*C*_) (see Section 2.2.2).

**Table 3:** Characteristic parameters of the continuous models

**Characteristic parameters of the *L*-model**	**A_1_**	**A_2_**	***L*_*EXC *_(*mm*)**	***p*-value**
Best fit values	4.1	0.6	2.5	0.03
**Characteristic parameters of the *HLD*-model**	**B_1_**	**B_2_**	***H *(*mm*)**	***p*-value**
Best fit values	5.8	0.1	0.7	< 0.0001
**Characteristic parameters of the *WS*-model**	**K_1_**	**K_2_**	***H, L*_*EXC*_**	***p*-value**
Best fit values	5.4	0.4	*set*	0.001

Characteristic parameters of the continuous models defined at a system level of description that best reproduces the experimental observations. *L-model *considers the effect of the distance between the walls of the culturing device, and *L*_*EXC *_is the region near the walls of the culturing device where the propagation of the parasite is hindered; *HLD-model *considers the effect of the depth of the haematocrit layer, with *h *the horizontal subregion where the propagation of the parasite is not hindered; *WS-model *merges both models and aims to reproduce the culture behaviour for any geometric layout; *L*_*EXC *_and *h *have maintained their values from the best fits obtained with the *L- *and the *HLD- *models. The values for the parameters *K*_1 _and *K*_2 _have been set through the optimization of the likelihood of the model outcome to experimental data. Significance of the obtained results was assessed using the Kolmogorov-Smirnoff (*KS*) test; the values presented show the probability of obtaining better results assuming the null hypothesis.

### Effect of the haematocrit layer depth (*HLD *model)

The KW test showed that data obtained from *W *and *B *series come from the same distribution function with significance greater than 99.9%. Thus, all the measurements have been grouped in a single data sample. Average values are given for the data corresponding to similar geometric conditions, for each of the subgroups: {W3+B1}, {W4+B2+B3} and {W5+B4+B5}.

**Figure 4:**
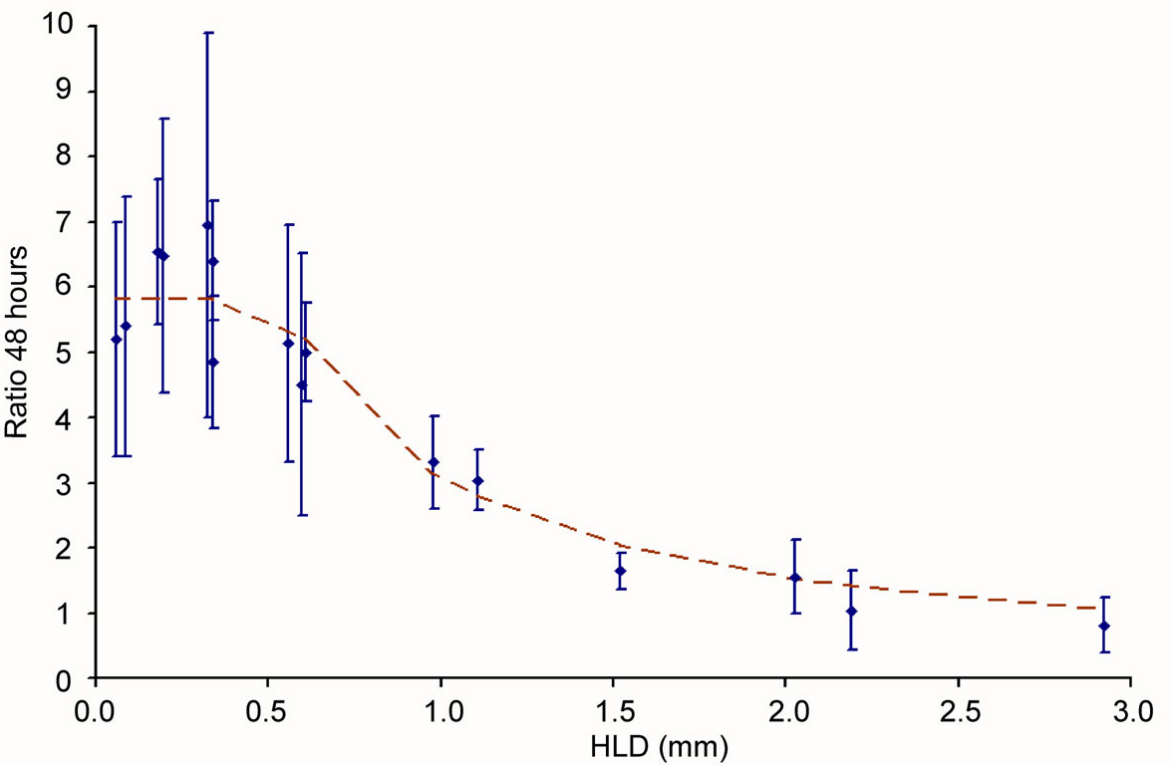
**Dependence of the parasitaemia on the haematocrit layer depth (*HLD*).** Dots with thin error bars represent the observed data. The dashed line denotes the best fit for the whole system model.

Experimental data show asymptotic inversely linear behaviour for large values of *HLD *(Figure 4). Optimum parasite development occurs in cultures with *HLD *between 0.18 *mm *and 0.34 *mm*. A simple system-level model that reproduces the observed behaviour consists in splitting the haematocrit layer into discrete regions that have different behaviours. The inversely linear decay for deep cultures is reproduced when the haematocrit layer is split into two horizontal regions: *HLD *= *h*_1 _+ *h*_2_. The first region has a fixed depth *h*_1 _= *h *and shows a high fixed infection multiplication ratio *B*_1_, while the infection spreads at a lower rate (*B*_2 _<*B*_1_) in the remaining part of the haematocrit layer: *h*_2 _= (*HLD*-h) (Figure 3b). Let *B*_1 _and *B*_2 _be the multiplication ratio per infection cycle in each of the above mentioned subregions, and *HLD *the total depth of the haematocrit layer. The average infection growth ratio at 48 h, *R*_48_(*HLD*) is then given by Equation 3:

(3) $$R_{48}(HLD)=\{\begin{array}{cc} B_{\text{2}}+(B_{\text{1}}-B_{\text{2}})\cdot \frac{h}{HLD} & if\;HLD>h\\ B_{\text{2}} & if\;HLD<h \end{array}$$

This behaviour can be theoretically supported by the consideration of local diffusive limitations of relevant solutes through the haematocrit layer. For instance, parasite development could be optimal in the vicinity of the interface with the free culturing medium, and hindered in 'deep regions' of the haematocrit layer due to the local scarcity of glucose, or to the excess of harmful metabolic waste products. The parameters that define the curve that best fits with the experimental data set are shown in Table 3.

The parameters have been estimated using a numerical approximation, and their likelihood given the observations has been checked using both the Kolmogorov-Smirnoff (*KS*) test and the chi-squared test (c^2^). The former statistic has been taken as the reference for the statistical significance of likelihood, because it implies fewer restrictions on both data sets and provides a smaller value for the degree of confidence of the results.

### Whole-system model that accounts for *HLD *and *L *together (*WS *model)

The models presented in sections 3.1 and 3.2 can be merged into a system-level representation of the whole culture (Figure 3c). The evolution of the culture as a function of the geometric variables *L *and *HDL *is shown in Equation 4:

(4) $$R_{48}(L,HLD)=\{\begin{array}{cc} K_{\text{2}}+(K_{\text{1}}-K_{\text{2}})\cdot \frac{h}{HLD}\cdot (\text{1}-\frac{{\text{2L}}_{\text{EXC}}}{L}) & if\;HLD>h\\ K_{\text{1}}\frac{{\text{2L}}_{EXC}}{L}+K_{2}(\text{1}-\frac{{\text{2L}}_{\text{EXC}}}{L}) & if\;HLD<h \end{array}$$

**Figure 5:**
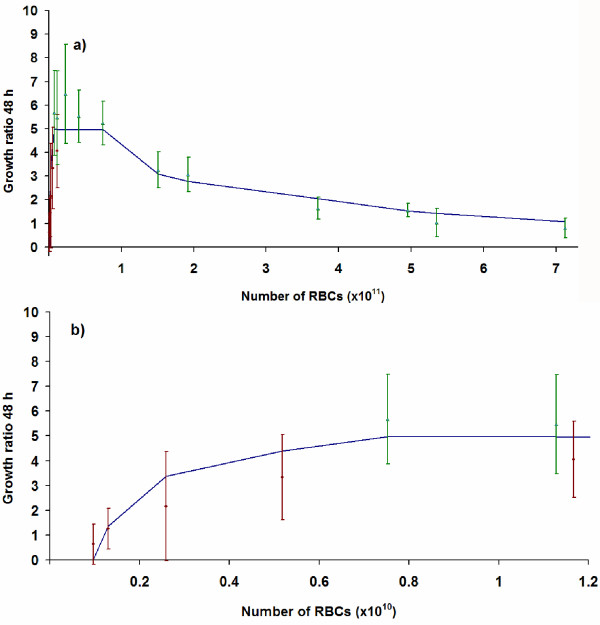
**Dependence of the parasite growth ratio both on the haematocrit layer depth (*HLD*) and width (L), represented as the dependence on the total number of cultured RBCs.** Dots with error bars represent the observed data from *P *trials. Triangles with error bars represent the averaged observed values from *W *and *B *trials. The solid line represents the best fit values provided by the WS model. a) Experimental data sets from *W *and *B *trials on the whole. b) Detail for trials with small culture volumes.

The values for *L*_*EXC *_and *h *have been adopted from the best fit values described above. The other parameters defined in this framework (*K*_1 _and *K*_2_) refer to the growth ratios in the subregions with high and low parasite propagation, respectively, and are calculated from the best fit to experimental data. The obtained values are presented in Table 3. A graphical comparison between the theoretical predictions and the experimental behaviour is shown in Figure 5. The KS test applied to expected and observed data sets gives a confidence between 90% and 95% for the proposed model.

This merging allows checking of the consistency of the models proposed above both with each other and with the experimental observations. It enables the specification of the most appropriate geometric conditions for the static *in vitro *cultures of *P. falciparum *infected erythrocytes.

### IBM that reproduces the experimental behaviour

Once the model for the whole system has been built, the observed behaviour can be tackled through a bottom-up IBM approach, so that the rules governing the system as a whole from the set of rules governing the individual parasites and RBCs at a cellular level can be reproduced. Such a model has the general structure that was presented by Ferrer *et al *[12], and is subject to the constraints imposed by the above presented whole-system model, i.e., it splits the simulation space into two horizontal sub-regions (layers 1 and 2, respectively; Figure 3b) with different probabilities of infection per parasite, and it permits assessment of the effect of placing a vertical wall on one of the side boundaries of the simulation grid.

**Figure 6:**
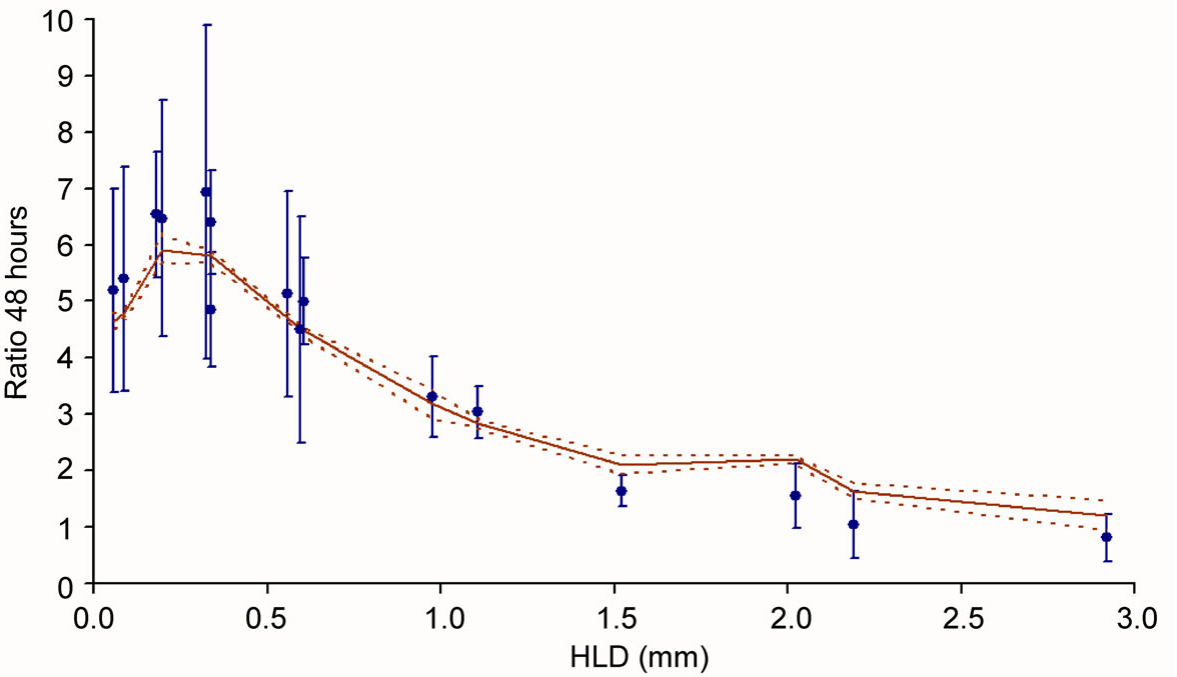
**Dependence of the parasite growth ratio on the haematocrit layer depth (*HLD*).** Dots with error bars represent the observed data. The solid line denotes the best fit for the Individual-Based Model simulation outcomes. Dashed lines represent the deviation observed for four simulation runs of each of the observed points.

The IBM is used to tackle solely the geometric constraints on parasite proliferation at a cellular level, so just a few parameters from the general model have been taken into account, and the remaining ones have been held to fixed values. The parameters of the model that have been optimized to fit the experimental data are the maximum probability of individual infection per time step once the extracellular parasite is in a spatial cell occupied by a healthy RBC, defined for each of the subregions (*p*_*inf*_(1) and *p*_*inf*_(2)), and the parameter that governs the spreading of the extracellular parasite through the haematocrit layer: the probability of a spatial cell shift per time step (*p*_*fall*_). These parameters have been estimated through numerical approximation, using the values that provided the simulation outcomes that best fit with the macroscopic observations. They are presented in Table 4. A graphical comparison between the IBM model and the whole-system model is shown in Figure 6. It must be stressed that this estimation of the optimal values has been carried out through a coarse exploration of the space of parameters; better fits could be found by the recursive refinement of the optimization protocol. Such improvement of the values at a cellular level has not been made because parameters such as *p*_*inf *_concern specific characteristics of the parasite strain and blood sample, and thus do not provide general insight concerning the culture system. The likelihood of the simulation results, given the experimental observations, has been checked using both the Kolmogorov-Smirnoff (*KS*) test and the chi-squared test (c^2^). Again, the former statistic has been taken as the reference for the statistical significance of the likelihood, because it implies fewer restrictions on both data sets and it provides a smaller value for the degree of confidence of the results.

**Table 4:** Characteristic parameters of the IBM-model

**Characteristic parameters of the IBM-model**	***p*_*inf*_(1)**	***p*_*inf*_(2)**	***p*_*fall*_**
Best fit values	0.85	0.1	0.05

Characteristic parameters of the Individual-Based Model that best reproduce the experimental observations. The values for the parameters *p*_*inf*_(1), *p*_*inf*_(2) and *p*_*fall *_were set through the optimization of the likelihood of the model outcome to experimental data. Significance of the obtained results was assessed using the Kolmogorov-Smirnoff (*KS*) test; the presented values show the probability of obtaining better results with the null hypothesis.

## Discussion

The systematic study of different macroscopic culturing conditions has allowed for the building of a quite simple model which is compatible with the experimental observations and which may account for some as yet poorly understood phenomena.

Some conclusions may be drawn from the experimental results, assuming the whole-system model:

1. Cell-cell interactions such as erythrocyte aggregateness and rosette formation around parasitized cells can be accounted for as average intercellular binding energy that determines the macroscopic shape of the haematocrit layer in the *in vitro *cultivation of *P. falciparum*-infected erythrocytes. Under custom culturing conditions, the haematocrit layer can be considered as a flat film, but such depiction is not valid when the haematocrit volume decreases. At small volumes of haematocrit the intracellular binding energy (which can be tackled as a surface tension on the haematocrit boundaries) is comparable to gravitational energy, so the haematocrit must be regarded as a sessile drop at the macroscopic scale. Different haematocrit shapes are observed depending on the material of the culturing device.

2. Geometric conditions of the culture systems at a macroscopic level of description play an important role in parasite development. Most appropriate dimensions of haematocrit layer depth (*HLD*) range from 0.18 mm to 0.34 mm. The spread of the infection is strongly hindered when *HLD *> 1 *mm*. According to this model, the spread of the infection is strongly hindered by short distances between walls (*L*), and cultures are unviable when *L *<*L*_*EXC *_= 2.5 *mm*. By extrapolating Equation 4, it is deduced that the effect of the exclusion region can be overlooked when *L *> 2 *cm *with more than 95% confidence. Effective parasite development takes place solely in a limited region of the haematocrit layer. One of the possible culturing scenarios shows the area of parasite proliferation covering solely the haematocrit upper surface (properly speaking, the interface between the haematocrit layer and the free culturing medium), excluding the boundaries in contact with the walls of the culturing device.

A bottom-up approach can be used to check the validity and consistency of the system-level model (WS model). This may provide justification for splitting the haematocrit layer into two subregions due to the diffusive limitations of the substrate through the haematocrit, and the consequent formation of gradients of concentration that may hinder the viability of the IRBCs. Such an approach also enables specific study and treatment of the relevant processes occurring at the scale of the parasite. The macroscopic subregions of different parasite proliferations (layers 1 and 2 in Figure 3) are externally imposed on the IBM as modifications on the parasite proliferation at a local level. Some additional conclusions may be drawn from analysis of the IBM:

all authors have read and approved the final manuscript.

3. The maximum threshold for the growth ratio at the zone of high parasite proliferation is geometrically fixed. The local multiplication of the parasite is not enough to ensure its propagation; a minimum spreading range is also required. This is shown by the model when increasing the maximum probability of individual infection (*p*_*inf*_) above a certain threshold value and noting that this does not increase the infection growth ratio. Indeed, allowing a greater diffusion rate for the extracellular parasite through the haematocrit layer (*p*_*fall*_) does increase the global growth ratio over the previous maximum threshold. The definition of the spreading range strongly affects the system-level outcome behaviour. In particular, if merozoites are allowed to propagate fast enough (*p*_*fall *_> 0.2 per time step), the decay in the infection with (*HLD*) is hindered.

4. The observed reduction in the average *R*_48 _for very thin haematocrit layers (unfeasibility of the harvest when *HLD *< 0.04 *mm*, and observable effects on cultures with *HLD *< 0.2 *mm*) can be reproduced with the IBM even though it has not been introduced as an external input on the rules governing the model. This is an example of an emergent behaviour that arises from the local interactions among individuals, and it is caused by the effect of the bottom boundary of the system on the propagation of the parasite. Such behaviour can be related to a hindering effect caused by the bottom of the culturing devices on real experimental systems.

## Conclusion

The present study provides some hints for the predictive understanding of *P. falciparum*-infected erythrocytes static *in vitro *cultures under different geometric constructions. A systematic study has been carried out examining a wide range of geometric configurations, and the observed behaviour has been formalized in a system-level phenomenological model.

The theoretical analysis of the phenomenological laws has been carried out by means of a bottom-up approach considering solely the geometric limitations on the parasite propagation. However, many other factors should be taken into consideration in order to obtain a broader depiction of the system.

## Competing interests

The authors declare that they have no competing interests.

## Authors' contributions

MDR, JMV, EH and DG performed the systematic experimental trials; CP, JMV, JF and DL built the system-level and IBM models; and JF, CP, DL and JV carried out the IBM simulations and analysed the results. All authors have read and approved the final manuscript.
